# Supplementary material for: Internal radiation exposure from 137Cs and its association with the dietary habits of residents from areas affected by the Chernobyl nuclear accident, Ukraine: 2016–2018
Source: PLoS One. 2023 Sep 15;18(9):e0291498. doi: 10.1371/journal.pone.0291498 (PMC10503735; doi:10.1371/journal.pone.0291498)
Supplement: Attachment — Submitted filename: Response to reviewers.doc [file pone.0291498.s003.doc]

July 7, 2023

Dear Dr. Hetal Bhadricha,

Thank you for letting us know the Decision: ‘PLOS ONE Decision: PONE-D-23-19852’. We also appreciate the time and effort you have handled our paper. We apologize our misinterpret and mistakes in our previous submission. We can definitely solve this issue. We also hope that our edits and the responses we provide below satisfactorily address all the issues and concerns you have noted.

**Comment of Decision Letter**

Based on our evaluation of your manuscript and the information you provided, we do not feel that your submission meets our ethical requirements for human subjects research submissions. PLOS ONE requires that research meets all applicable standards for the ethics of experimentation and research integrity (http://journals.plos.org/plosone/s/human-subjects-research). We reserve the right to reject any submission that does not meet our internal ethical standards, which in some cases are more stringent than local ethical standards.

We recognize that you obtained ethical approval from your local ethics committee after you began your study: your ethics approval document is dated 31 March 2021, while participants were recruited beginning in July 2016. You also state that informed consent was obtained from all participants, which indicates that the participant's data were collected prospectively for research purposes. For human subjects research, PLOS ONE requires that authors obtain ethics committee approval before beginning the study, and we do not accept retrospective ethical approval.

**Response.** We can fully address these comments because we obtained ethical approval from our institution’s ethics committee from the beginning of this study. We submitted full ethics approval documents as ‘other’ file.

With the incorporation of these amendments to our final manuscript, we hereby resubmit our manuscript for a secondary evaluation. We hope that the revised version of our paper is now suitable for publication in *PLoS ONE*, and we look forward to hearing from you at your earliest convenience.

Sincerely,

Naomi Hayashida, M.D., PhD.

Professor of Division of Strategic Collaborative Research, Center for Promotion of Collaborative Research on Radiation and Environment Health Effects, Atomic Bomb Disease Institute, Nagasaki University

1-12-4 Sakamoto, Nagasaki 852-8523, JAPAN

TEL: +81-95-819-8507

FAX: +81-95-819-8508

E-mail: [naomin@nagasaki-u.ac.jp](mailto:naomin@nagasaki-u.ac.jp)
