# Supplementary material for: Internal radiation exposure from 137Cs and its association with the dietary habits of residents from areas affected by the Chernobyl nuclear accident, Ukraine: 2016–2018
Source: PLoS One. 2023 Sep 15;18(9):e0291498. doi: 10.1371/journal.pone.0291498 (PMC10503735; doi:10.1371/journal.pone.0291498)
Supplement: Attachment — Submitted filename: Response_to_reviewers.docx [file pone.0291498.s004.docx]

August 21, 2023

Tim A. Mousseau

Academic Editor

PLOS ONE

Dear Mr. Mousseau,

Thank you for inviting us to resubmit a revised draft of our manuscript entitled, ‘Internal radiation exposure from ^137^Cs and its association with the dietary habits of residents from areas affected by the Chernobyl nuclear accident, Ukraine: 2016–2018’ [PONE-D-23-21136] to *PLoS ONE*. We also appreciate the time and effort you and the reviewers have dedicated to providing insightful comments on ways to strengthen our paper. Thus, we are delighted to resubmit our article for further consideration. We have incorporated the changes that reflect the detailed suggestions you have so graciously provided. We also hope that our edits and the responses we provide below, satisfactorily address all the issues and concerns.

To facilitate your review of our revisions, the following is a point-by-point response to the questions and comments delivered in your E-mail dated 27 July 2023.

- **Editor**

We appreciate your earnest comments and valuable suggestions that have notably reinforced the structure and improved the comprehension of our manuscript. We have considered all the comments and suggestions that were submitted and responded respectively as provided below.

**Comment 1.** Please ensure that your manuscript meets PLOS ONE’s style requirements, including those for file naming.

**Response.** We made sure that the manuscript meets all of PLOS ONE’s style requirements and as well as the file naming. (Links for templates [1](https://journals.plos.org/plosone/s/file?id=wjVg/PLOSOne_formatting_sample_main_body.pdf) and [2](https://journals.plos.org/plosone/s/file?id=ba62/PLOSOne_formatting_sample_title_authors_affiliations.pdf))

**Comment 2.** Please include a complete copy of PLOS’ questionnaire on inclusivity in global research in your revised manuscript. Our policy for research in this area aims to improve transparency in the reporting of research performed outside of researchers’ own country or community. The policy applies to researchers who have travelled to a different country to conduct research, research with Indigenous populations or their lands, and research on cultural artefacts. The questionnaire can also be requested at the journal’s discretion for any other submissions, even if these conditions are not met. Please find more information on the policy and a link to download a blank copy of the questionnaire here: <https://journals.plos.org/plosone/s/best-practices-in-research-reporting>. Please upload a completed version of your questionnaire as Supporting Information when you resubmit your manuscript.

**Response.** We completed the questionnaire on inclusivity and uploaded with our revised manuscript under the title of file “S1 Questionnaire”.

**Comment 3.** Please review your reference list to ensure that it is complete and correct. If you have cited papers that have been retracted, please include the rationale for doing so in the manuscript text or remove these references and replace them with relevant current references. Any changes to the reference list should be mentioned in the rebuttal letter that accompanies your revised manuscript. If you need to cite a retracted article, indicate the article’s retracted status in the References list and also include a citation and full reference for the retraction notice.

**Response.** We have ensured that the reference list is complete and correct. We did not find record of retraction of any paper we listed in the reference that are part of our manuscript.

- **Reviewer #1:**

We are grateful to reviewer #1 for the critical comments and useful suggestions that have helped us to improve our paper considerably. As indicated in the responses that follow, we have taken all these comments and suggestions into account in the revised version of our paper.

**Comment 1.** The Abstract only presents qualitative research methods, results and conclusions. At the very least, the years of investigation and the key conclusions should be "quantitatively" described.

**Response.** We have revised the content of the abstract and added quantitative information regarding the research details and conclusion.

**Line 28-30:** *We screened 1,612 participants, from July 2016 to February 2018 for internal radioactivity, using whole-body counter at Korosten Medical Center and surveyed their background and intake habits.*

**Line 38-42:** *Radioactivity detected in the mushroom-berry and only-mushroom group were 8.6 and 9.2 Bq/kg, respectively. The lowest and highest intake frequency showed a radioactivity of 2.4 and 7.5 Bq/kg, respectively. Radioactivity in the winter season was significantly higher than that in other seasons. In conclusion, our study revealed that internal radioactivity varies depending on the type of food, intake frequency, and season.*

**Comment 2.** I have some questions about the detectable limits. The WBC used in the study is described as a type with a 7.6 cm (3-inch, i think) NaI detector, Aloka, but it maybe probably a chair type. If this is the case, please indicate specifically how the detection limit (or MDA) of 270 Bq/body was calculated, using the 3sigma or Currie's method.

**Response.** The Aloka Whole Body Counter (WBC) is a chair-type equipment. We modified the sentence describing the WBC, so the reader can have a better understanding. The minimum detectable activity of WBC is set by the producer of the equipment. Therefore, we did not adjust MDA nor calculated it.

**Line 141-142:** *It is chair-type WBC with an inbuilt 7.6-cm-diameter NaI (TI) detector, where the examinee is supposed to place his/her abdomen.*

**Comment 3.** Supplemental information indicates that there is individual with extremely high radioactivity levels of 237 Bq/kg (29 years old, see attaached image file). Statistics are important, but discussing this individual case is crucial to understanding the reality of the exposure. I would like to know the reasons why authors did not mention about this case.

**Response.** Our research was designed mainly to focus on the general population; thus, we primarily examined all participants as groups that are represented on average. However, we agree that some exceptional cases should be observed and discussed in detail. We incorporated a paragraph about this particular case that was pointed out by the reviewer,

in the discussion part of our manuscript,

**Line 387-396:** *We also examined the individual that outstood from other participants with the highest internal exposure, measured with 237 Bq/kg. According to answers to the questionnaire, this female individual had consumed berries and mushrooms 3 times a week and had gotten measurement on WBC in winter. Considering the results of our research, this participant belongs to the groups that were identified with highest level of internal contamination. Most of those who consumed mushroom and berries 3 times a week were measured with the highest level of radioactivity in body. Moreover, people who have been measured in winter, showed the highest level of body contamination. Therefore, most likely this participant had consumed highly contaminated forest foodstuff, either dried or bottled, just few days before measurement on the WBC.*

**Comment 4.** L150 modify "0.0025 msv/y/Bq/kg" to "0.0025 mSv/y/Bq/kg".

**Response.** We modified the description above as suggested.

- **Reviewer #2:**

We are grateful to reviewer #2 for the critical comments and useful suggestions that have helped us to improve our paper considerably. As indicated in the responses that follow, we have taken all these comments and suggestions into account in the revised version of our paper.

**Comment 1.** The authors have focused on beta emitters only. In the first few days iodine was the biggest problem, indeed; but during the accident extremely high amounts of other nuclides were released, including alpha-emitters which ionize the body in much bigger level. Thus, the information about iodine or Cs-134 (Introduction) seems to be less important. In my opinion the authors must add the information about alpha emitters.

**Response.** We agree that iodine and Cs-134 are less important in terms of ionizing effect on the body compared to that of alpha-emitters; however, our intention by mentioning them was to provide an overall picture of radioactive materials released from CNPP, particularly taking into account the amount of major radionuclides released. Also, this additional information was provided for those who have limited understanding about radioactive materials and their effects.

**Comment 2.** At the moment, the amount of Cs-137 is two times smaller, while most of alpha emitters are still present in the environment in unchanged quantity. It is hard to measure them, but they cause the highest ionizing effect in the body.

**Response.** We admit the fact that alpha emitters are still present in the environment, probably in unchanged quantity. However, the main goal of our research was Cs-137 and its chronic effect on the body of those living in contaminated areas. Even now, after several decades, Cs-137 persists in the environment in larger amounts despite its ionizing effect being halved owing to half-life longevity. Cs-137 is still being detected in people from contaminated areas, and it is also easily measurable and traceable.

**Comment 2.** The studies were well planned and organized, the analysed group is quite impressive, the analysis to the foodstuff types (the main sources of Cs-137) was done and presented clearly. The figures should be improved, their quality is insufficient. After small remarks the manuscript is worth publishing in PLOS One.

**Response.**

We appreciate all the suggestions listed above and will certainly take into consideration in our future research projects. We made some minor modifications to our figures, as suggested.

With the incorporation of these amendments to our final manuscript, we hereby resubmit our manuscript for a third evaluation. We hope that the revised version of our paper is now suitable for publication in *PLoS ONE*, and we look forward to hearing from you at your earliest convenience.

Sincerely,

Naomi Hayashida, M.D., PhD.

Professor of Division of Strategic Collaborative Research, Center for Promotion of Collaborative Research on Radiation and Environment Health Effects, Atomic Bomb Disease Institute, Nagasaki University

1-12-4 Sakamoto, Nagasaki 852-8523, JAPAN

TEL: +81-95-819-8507

FAX: +81-95-819-8508

E-mail: [naomin@nagasaki-u.ac.jp](mailto:naomin@nagasaki-u.ac.jp)
